# Endotracheal administration for intraoperative acute massive pulmonary embolism during laparoscopic hepatectomy: A case report: Retraction

**DOI:** 10.1097/MD.0000000000021978

**Published:** 2020-08-14

The article “Endotracheal administration for intraoperative acute massive pulmonary embolism during laparoscopic hepatectomy”^[1]^ which appeared in Volume 99, Issue 3 of *Medicine* is being retracted due to concerns regarding unsound scientific management of the reported case.
